# Does taking an angiotensin inhibitor increase the risk for COVID-19? – a systematic review and meta-analysis

**DOI:** 10.18632/aging.202902

**Published:** 2021-04-22

**Authors:** Zheng Ma, Mei-Ping Wang, Lian Liu, Shuang Yu, Tian-Ran Wu, Lei Zhao, Ye-Ping Zhang, Hai-Feng Liang, Xin-Chun Yang

**Affiliations:** 1 Heart Center and Beijing Key Laboratory of Hypertension, Beijing Chaoyang Hospital, Capital Medical University, Chaoyang 100020, Beijing, China; 2 Department of Epidemiology and Health Statistics, School of Public Health, Capital Medical University, Fengtai 100054, Beijing, China; 3 Department of Interventional Neuroradiology, Beijing Tiantan Hospital, Capital Medical University, Fengtai 100070, Beijing, China; 4 Yuetan Community Health Service Center, Fuxing Hospital, Capital Medical University, Xicheng 100045, Beijing, China; 5 Monash Medical Centre, Clayton, VIC 3168, Australia; 6 Heart Center, Fuxing Hospital, Capital Medical University, Xicheng 100038, Beijing, China

**Keywords:** coronavirus disease 2019, COVID-19, angiotensin-converting enzyme 2, angiotensin converting enzyme inhibitors, angiotensin receptor blockers

## Abstract

Because SARS-COV2 entry into cells is dependent on angiotensin converting enzyme 2 (ACE2) and angiotensin converting enzyme inhibitors (ACEIs) or angiotensin receptor blockers (ARBs) increase ACE2 activity, the safety of ACEI/ARB usage during the coronavirus disease 2019 (COVID-19) pandemic is a controversial topic. To address that issue, we performed a meta-analysis following The Preferred Reporting Items for Systematic Reviews and Meta-Analyses guidelines. Searches of the Embase, MEDLINE, PubMed, and Cochrane Library databases identified 16 case-control studies examining the effect of ACEI/ARB on the incidence of COVID-19 and its severity. ACEI/ARB usage was associated with an increased risk of COVID-19 morbidity (odds ratio (OR) 1.20, 95% confidence interval (CI) 1.07-1.33, P=0.001) among the general population but not in a hypertensive population (OR 1.05, 95% CI 0.90-1.21, P=0.553). ACEI/ARB usage was not associated with an increased risk of COVID-19 morbidity (coefficient 1.00, 95% CI 1.00-1.00, P=0.660) when we adjusted for hypertension in the general population. ACEI/ARB usage was also not associated with an increased risk of severe illness (OR 0.90, 95%CI 0.55-1.47, P=0.664) or mortality (OR 1.43, 95%CI 0.97-2.10, P=0.070) in COVID-19 patients. Our meta-analysis revealed that ACEI/ARB usage was not associated with either the increased risk of SARS-COV2 infection or the adverse outcomes in COVID-19 patients.

## INTRODUCTION

Coronavirus disease 2019 (COVID-19) has become the most devastating infectious disease caused by a coronavirus since the outbreak of severe acute respiratory syndrome in 2003 [1, 2]. The lack of knowledge about this novel coronavirus, named severe acute respiratory syndrome coronavirus 2 (SARS-COV2), has hindered the effective protection of especially vulnerable populations, as well as treatment for all patients [3, 4]. Recently, however, it was reported that entry of SARS-COV2 into host cells is dependent on angiotensin converting enzyme 2 (ACE2) [5]. This finding opened a new avenue for treatment of the disease, but was controversial in the cardiovascular field. It has been well established that ACE2 is an important member of renin angiotensin system (RAS) and participates in the protection of the cardiovascular system [6]. ACE inhibitors (ACEIs) and angiotensin receptor blockers (ARBs) are classical antihypertensive drugs that exert their effects via the RAS. Moreover, ACEIs and ARBs act in part by increasing cardiac ACE2 gene expression or inducing cardiac ACE2 activity [7], which raises the question, do these agents increase the risk of SARS-COV2 infection and/or worsen the prognosis of patients with COVID-19 [8–10]. Unfortunately, only a few retrospective studies have attempted to address these questions.

In the present meta-analysis, we used the results from published literature to try to answer 1) whether ACEI and/or ARB are associated with the risk of SARS-COV2 infection and 2) whether they are associated with adverse outcomes in patients with COVID-19.

## RESULTS

**Figure 1:**
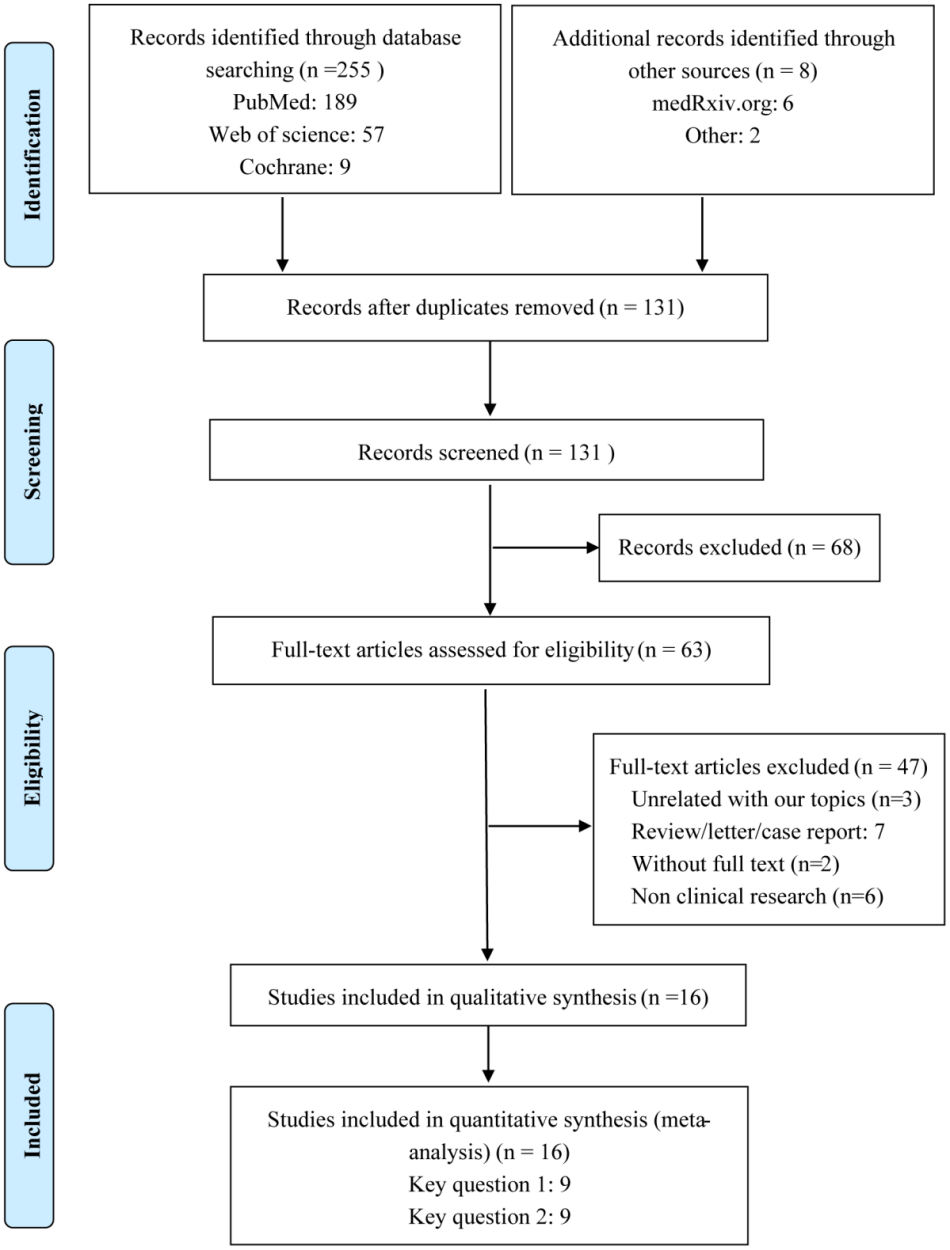
Flow diagram of the process for identification of included studies.

A flow diagram of the selection of included studies is shown in Figure 1. A total of 16 studies of COVID-19 conducted in China, Italy, USA, Spain, and Israel satisfied the inclusion criteria for this meta-analysis. These included 8 studies assessing the correlation between ACEI/ARB and the incidence of COVID-19 and 9 studies analyzing the relationship between ACEI/ARB and adverse outcomes in patients with COVID-19 [11–26] (Supplementary Table 2). Participants in 9 studies were from the general population, while the participants in the other 7 were hypertensive population (Supplementary Table 2). Methodological assessment of the included studies using NOS criteria revealed that 3 studies were of low quality and 13 were of high quality (Supplementary Table 1). Ultimately, a total of 116,111 individuals were included in our meta-analysis. The main clinal features of the general population are shown in Supplementary Table 2. The overall pooled prevalence of hypertension was 46.5% (95% CI 34.1%–58.9%), calculated using a random-effects model (P < 0.001, I^2^ = 98.0%) (Supplementary Figure 2). The overall rate of ACEI/ARB usage was 27% (95% CI 19.0-35.0%) in the general population and 41.0% (95% CI 20.0-62.0%) in the hypertensive population (Supplementary Figure 3).

### Association between of ACEI/ARB usage and COVID-19 morbidity

**Figure 2:**
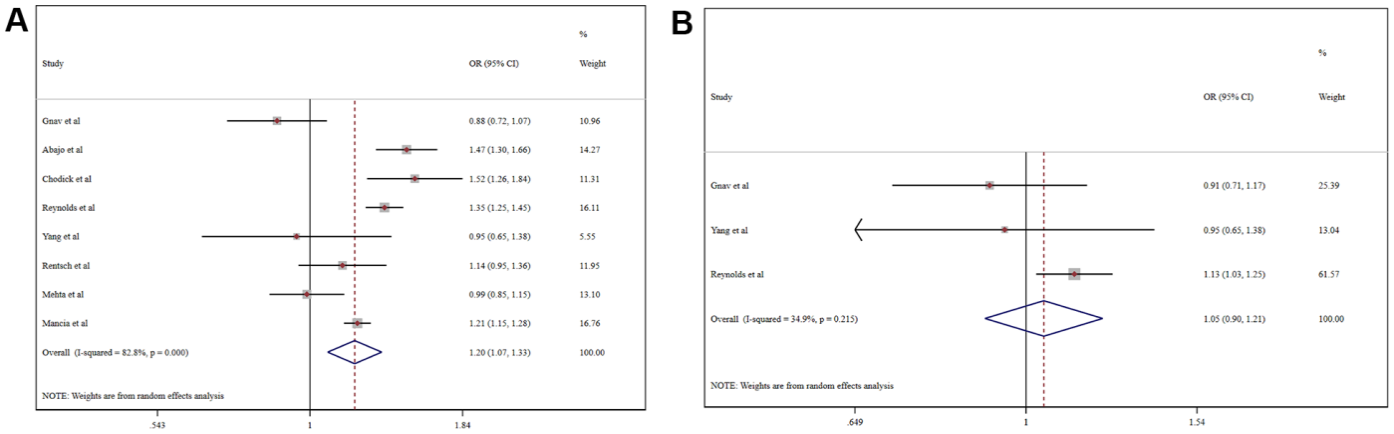
Forest plot of the correlation between ACEIs/ARBs and COVID-19 in the general population (**A**) and a hypertensive population (**B**).

A meta-analysis of the odds ratio (OR) for ACEI/ARB-related increases in the risk of COVID-19 morbidity is shown in Figure 2. Among the general population, the usage of ACEI/ARB was associated with higher risk of COVID-19 morbidity (OR = 1.20, 95% CI 1.07-1.33, P=0.001). Intriguingly, in the hypertensive population ACEI/ARB was not associated with increased COVID-19 morbidity (OR = 1.05, 95% CI, 0.90-1.21, P=0.553). Moreover, ACEI/ARB usage was not associated with the increased risk of COVID-19 morbidity in the general population when we adjusted for hypertension using meta-regression (coefficient 1.00, 95% CI 1.00-1.00, P=0.660).

### Association between ACEI/ARB usage and adverse outcomes in patients with COVID-19

**Figure 3:**
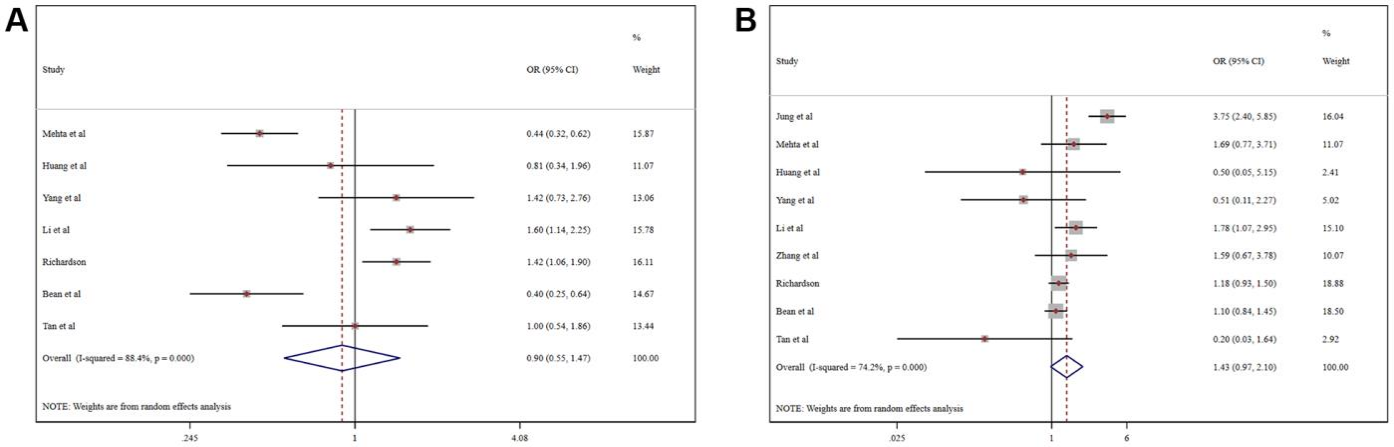
Forest plot of the correlation between ACEIs/ARBs and adverse outcomes in patients with COVID-19: (**A**) Severe COVID-19; (**B**) Mortality.

**Figure 4:**
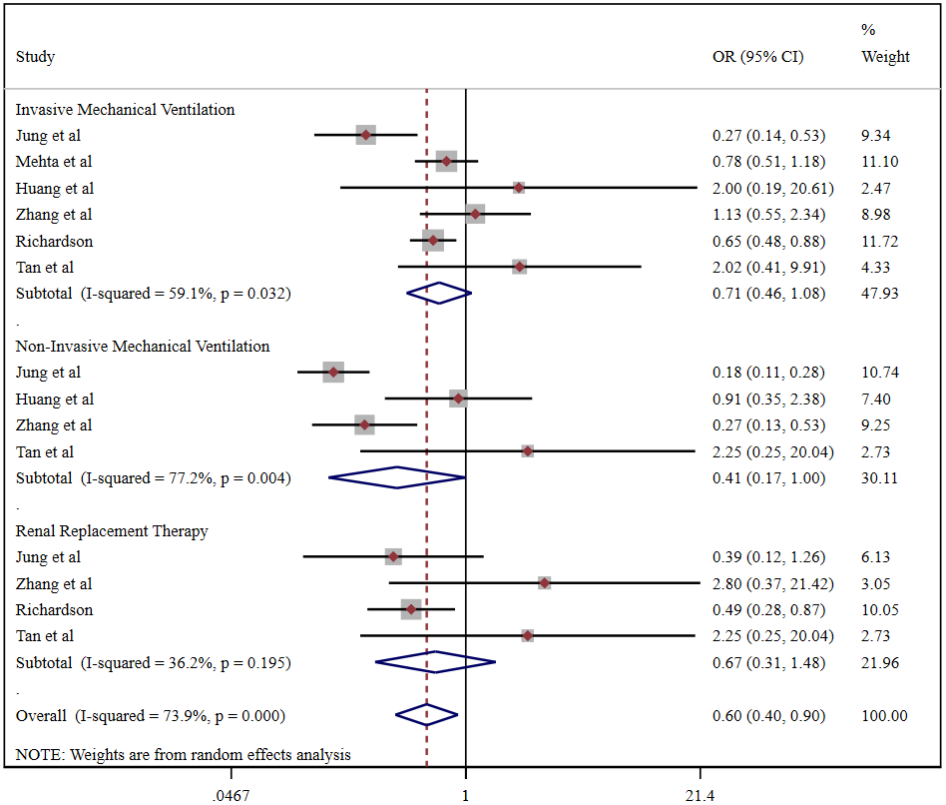
Forest plot of the correlation between ACEIs/ARBs and advanced life support in patients with COVID-19.

Pooled analysis revealed that the proportion of severe cases among COVID-19 patients was 41.5% (95%CI 26.8%-56.1%), overall mortality was 10.8% (95%CI 6.7%-14.9%), and the prevalence of total adverse outcomes (including severe cases and mortality) was 24.0% (95%CI 18.0%-29.0%) (Supplementary Figure 4). ACEI/ARB usage was not associated with either severity (OR = 0.90, 95%CI 0.55-1.47, P=0.664) or mortality (OR = 1.43, 95%CI 0.97-2.10, P=0.070) in COVID-19 patients (Figure 3). Patients taking an ACER/ARB did not have a higher rate of noninvasive mechanical ventilation, invasive mechanical ventilation or renal replacement therapy, and they had a low overall rate of advanced life support usage (OR = 0.60, 95%CI 0.40-0.90, P<0.001) (Figure 4).

## DISCUSSION

ACEIs and ARBs are frequently prescribed medications that are used by about 40% of hypertension patients in our meta-analysis. Given the high rate of ACEI or ARB usage and the role of ACE2 in the pathogenesis of COVID-19 [5], a key concern is whether use of an ACEI/ARB increases the risk of SARS-COV2 infection. The results from this meta-analysis indicate that that ACEI/ARB usage correlates positively COVID-19 morbidity in the general population, but that relationship is not detected in a hypertensive population. Moreover, when we adjusted for hypertension in the general population, the correlation between ACEI/ARB and morbidity from COVID-19 disappears. These results suggest the real risk factor for COVID-19 may be hypertension, not the ACEI or ARB used to treat it. Hypertension is a common comorbidity with a higher prevalence among COVID-19 patients than the general population [18]. Hypertension often coexists with other cardiovascular and metabolic diseases, especially in older people [27]. It is well known that chronic low-grade inflammation is a common feature of cardiovascular and metabolic diseases [28, 29], and the association between hypertension and the immune system is now accepted [30]. Changes in both innate and adaptive immunity that cause an imbalance between proinflammatory and anti-inflammatory processes contribute to pathological tissue repair and -remodeling not only in the cardiovascular system, but also in lung tissue [31]. Hypertension-associated low-grade inflammation and immunity imbalance may impair pulmonary circulation and reduce the immune barrier effect, which may, in turn, increase the risk of respiratory infection. Consequently, patients with hypertension may be more susceptible to SARS-COV2 infection than the general population, which is consistent with the results of our meta-analysis and observational studies [13, 18]. But if taking an ACEI or ARB increased the risk of SARS-COV2 infection, the correlation between ACEI/ARB and COVID-19 should be stronger in a hypertensive population than the general population. Our finding that this is not the case suggests ACEI/ARB usage is just a covariate of hypertension rather than a risk factor.

A second concern is whether ACEI/ARB usage is associated with adverse outcomes. We selected advanced life support, severe illness and mortality as end points. Our meta-analysis revealed that ACEI/ARB usage did not increase the risk of severe illness or death. Surprisingly, ACEI/ARB usage decreased the risk of needing advanced life support. ACEIs and ARBs are classes of drugs that effectively inhibit the RAS [32]. Their cardioprotective effects, which include anti-inflammatory, anti-oxidative stress and antifibrotic effects, have been confirmed in a large number of basic and clinical studies [33, 34]. In addition, the relationship between the RAS and pulmonary disease has also attracted attention [35]. Angiotensin II induces pulmonary vasoconstriction, exacerbates pulmonary fibrosis, promotes inflammation and enhances oxidative stress. ACEI/ARB-induced ACE2 expression may inhibit the adverse effects of angiotensin II in lung tissue. In a mouse model, for example, ACE2 protected against ARDS, while an ARB also protected against lung injury by blocking AT1 receptors [36]. Moreover, blocking AT1 receptors can suppress pulmonary fibrosis that increases the risk of severe COVID-19 [37]. Consequently, taking an ACEI or ARB may reduce the risk of severe COVID-19 and ARDS, thereby contributing to a lower usage rate of advanced life support. Whether or not this beneficial effect of ACEIs or ARBs actually exists [9, 38], we did not detect an association between ACEI or ARB usage and severe COVID-19 or mortality in our meta-analysis.

Although a meta-analysis based on randomized controlled trials (RCTs) represents the highest level of evidence, there have been only few RCTs designed to investigate the safety of ACEIs or ARBs for patients with cardiovascular disease during the COVID-19 pandemic. However, one recent study shed light on ramipril’s impact on COVID-19 risk in this vulnerable population [39]. The participants in that study came from The RASTAVI (Renin-Angiotensin System Blockade Benefits in Clinical Evolution and Ventricular Remodeling After Transcatheter Aortic Valve Implantation) trial and were randomly assigned to a ramipril or control group. The results showed that ramipril had no impact on the incidence or severity of COVID-19, which is in line with our results. Additional protocols from RCTs have also been published, and their results deserve attention [40–42]. Thus, our systematic review based on currently available large cohort studies will have clinical value until the results of additional well-designed RCTs with large sample sizes become available.

Our results reveal that ACEI or ARB usage was not associated with either the increased risk of SARS-COV2 infection or the pool prognosis of COVID-19. It is therefore unnecessary to discontinue use of an ACEI or ARB during the COVID-19 pandemic. Nonetheless, given the complexity of the interaction between RAS inhibitors and COVID-19, more in-depth studies to resolve existing controversies are required.

## MATERIALS AND METHODS

### Literature search

This meta-analysis followed the Preferred Reporting Items for Systematic Reviews and Meta-Analyses (PRISMA) statement guidelines [43]. Literature published in English and listed in the Embase, MEDLINE, PubMed, and/or Cochrane Library databases were searched by two independent reviewers from January 1 to August 15, 2020. The search terms ‘angiotensin converting enzyme inhibitor’, ‘angiotensin-converting enzyme inhibitor’, ‘ACEI’, ‘angiotensin receptor blocker’, ‘ARB’, ‘novel coronavirus’, ‘novel coronavirus 2019’, ‘2019nCoV’, ‘COVID-19’, ‘Wuhan coronavirus’, ‘Wuhan pneumonia’, ‘SARS-CoV-2’ and ‘Corona virus disease 2019’ were included in our search strategy. Any inconsistency between the reviewers was resolved by a third independent reviewer.

### Eligibility criteria

High-quality cohort and case-control studies were included in this systematic review and meta-analysis. Studies that reported the usage of an ACEI and/or ARB and SARS-cov-2 infection were eligible. Studies that reported an association between ACEI and/or ARB usage and the outcomes of COVID-19 patients were also included. Studies without adverse outcomes, including usage of advanced life support, severe COVID-19 and death, were excluded after reviewing Supplementary Materials.

### Data extraction

Two independent reviewers used a predetermined data collection table to extract relevant data. Any divergence between the reviewers was resolved by a third independent reviewer.

### Quality assessment

Two of the authors used the Newcastle-Ottawa Quality Assessment Scale (NOS) to independently assess the quality of observational studies [44].

### Outcomes of interest

The primary endpoints were case confirmation and adverse outcomes, including severe disease and death, in patents with COVID-19. The secondary outcome was usage of advanced life support by COVID-19 patients during their hospitalization.

### Statistical analysis

Meta-analysis was performed using Stata version 15.0 (StataCorp, College Station, TX, USA). Results were presented ORs and CIs on the basis of the Mantel-Haenszel random-effects model. Heterogeneity was evaluated with the I^2^ statistic. Values of I^2^ >50% were regarded as indicating considerable heterogeneity. For qualitative evaluation of publication bias toward the endpoint, funnel plots were used; for quantitative assessment, Egger’s linear regression test and Begg’s rank correlation test was used (Supplementary Figure 1). We conducted a sensitivity analysis of the endpoint through sequential removal of each study. Meta-regression analyses based on study-level covariates (age, sex, hypertension history) were conducted to explain any heterogeneity. We calculated the optimal information (sample) size to maintain a 2-sided type I error at 0.05 and a type II error at 0.20 (80% power), with a relative risk reduction of 25% and an incidence of 8.5% endpoint in the central arm.

## Supplementary Material

Supplementary Figures

Supplementary Table 1

Supplementary Table 2
