# Supplementary material for: Does taking an angiotensin inhibitor increase the risk for COVID-19? – a systematic review and meta-analysis
Source: Aging (Albany NY). 2021 Apr 22;13(8):10853–65. doi: 10.18632/aging.202902 (PMC8109111; doi:10.18632/aging.202902)
Supplement: Supplementary Figures [file aging-13-202902-s001.pdf]

SUPPLEMENTARY FIGURES

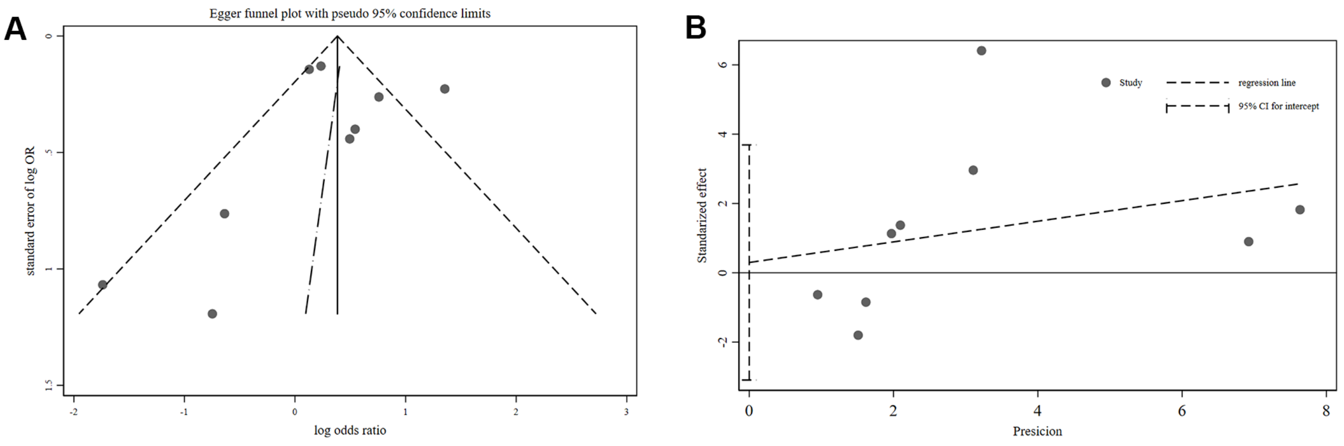

Supplementary Figure 1. Funnel plots for publication bias testing. (**A**) Egger's funnel plots of included studies. (**B**) Begg's funnel plots of included studies.

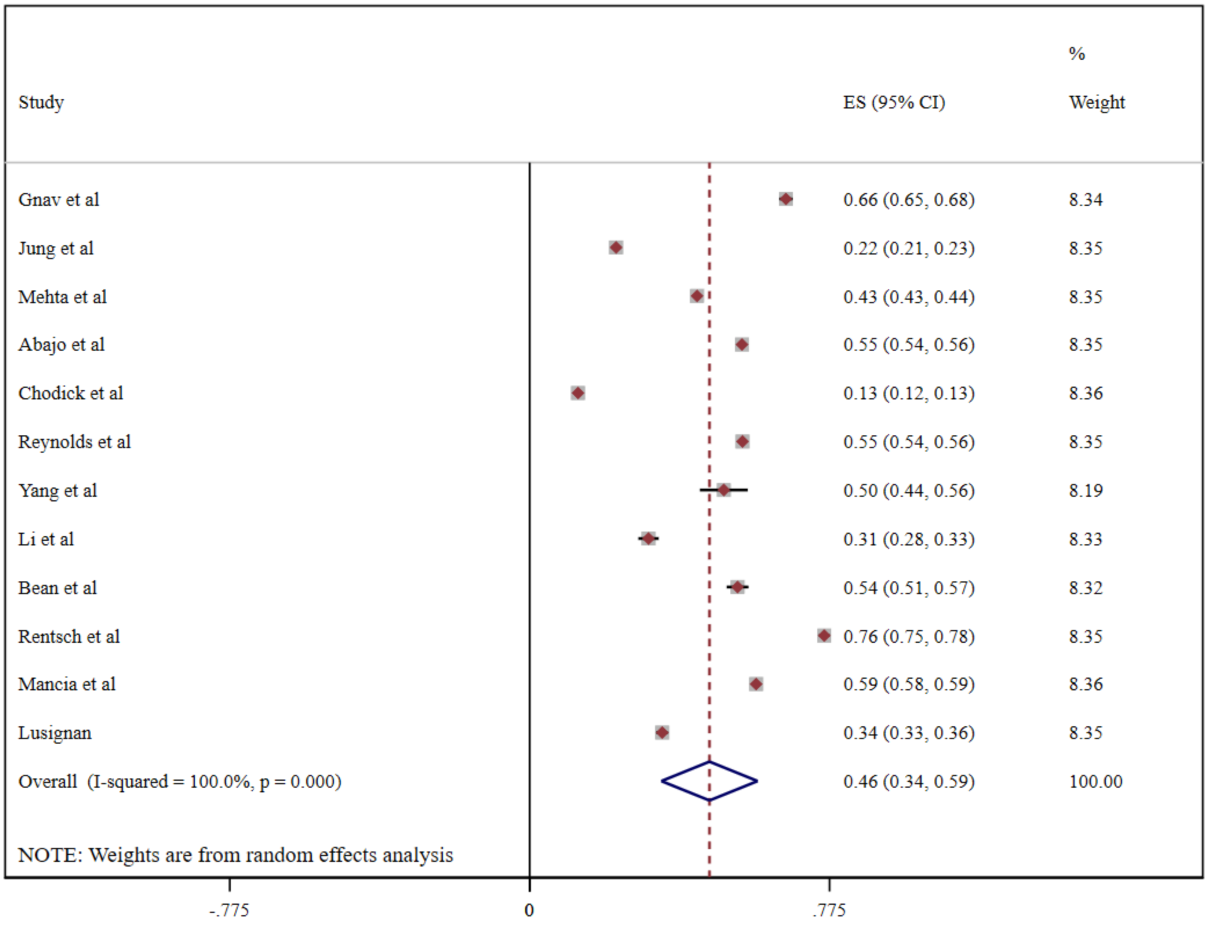

Supplementary Figure 2. Forest plot of the prevalence of hypertension in general population.

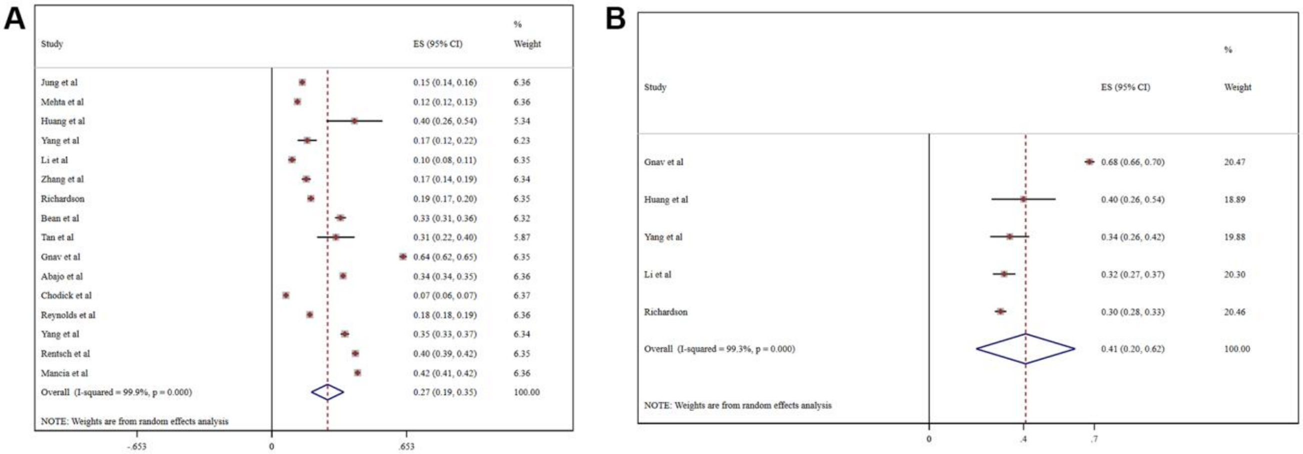

Supplementary Figure 3. Forest plot of the ACEI/ARB usage rate in the general population (**A**) and a hypertensive population (**B**).

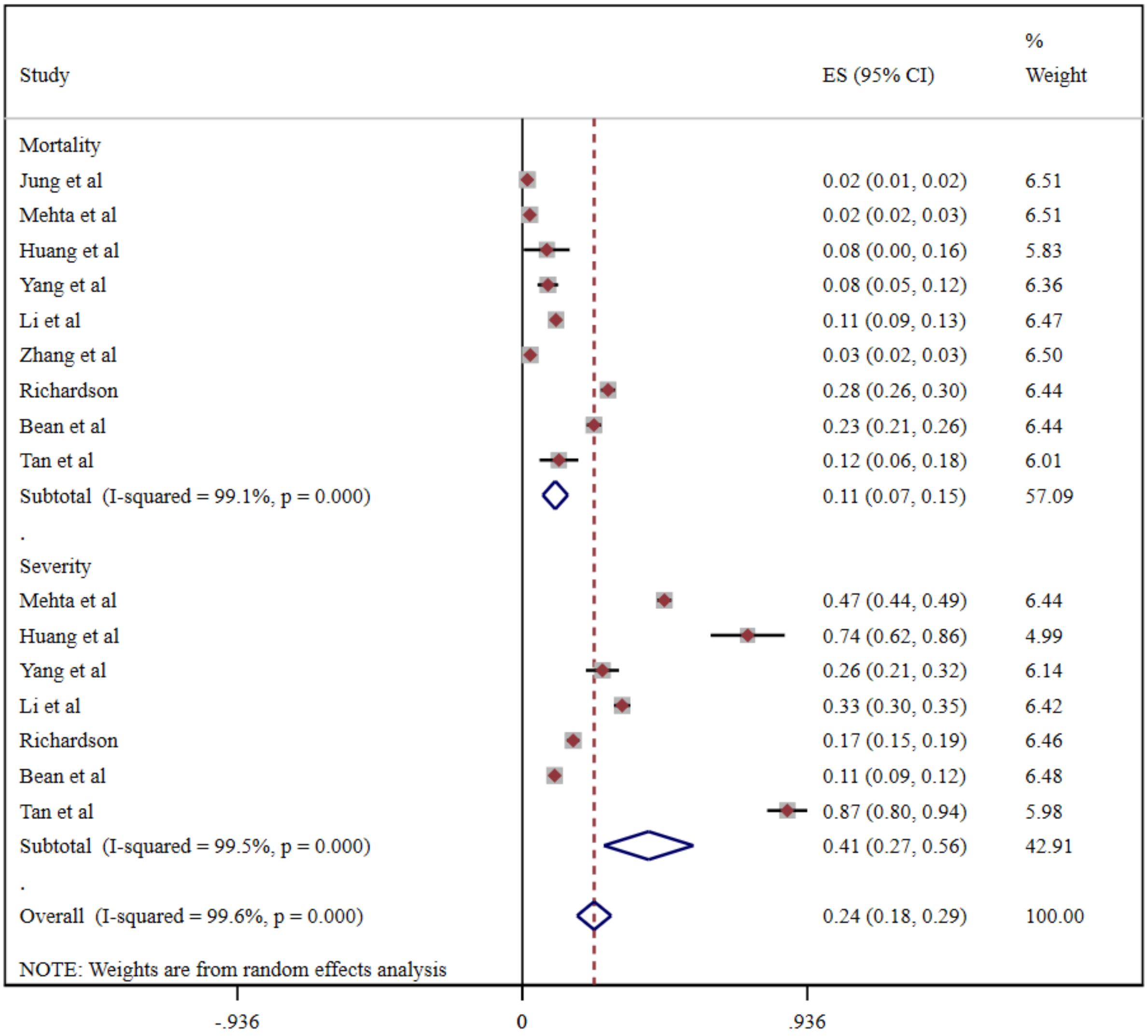

Supplementary Figure 4. Forest plot of the prevalence of adverse outcomes, including severe COVD-19 and mortality, in the general population.
