# Supplementary material for: Does taking an angiotensin inhibitor increase the risk for COVID-19? – a systematic review and meta-analysis
Source: Aging (Albany NY). 2021 Apr 22;13(8):10853–65. doi: 10.18632/aging.202902 (PMC8109111; doi:10.18632/aging.202902)
Supplement: Supplementary Table 1 [file aging-13-202902-s002.pdf]

Supplementary Table 1. Methodological assessment of included studies using the Newcastle-Ottawa Scales (NOS) for longitudinal studies.

Study	Selection				Comparability	outcome			Overall score and quality
	Representativeness of the sample	Sample size	Ascertainment of exposure	Non-respondents	Based on the design or analysis	Assessment of the outcome	Follow-up	Adequacy of follow up	
Gnavi R et al [11]	*	*	*	*	**	*	*	*	9 high
de Abajo FJ et al [12]	*	*	*	*	**	*	*	*	9 high
Chodick G et al [13]	*	*	*	*	*	*			6 low
Reynolds HR et al [14]	*	*	*	*	**	*	*	*	9 high
Yang G et al [15]	*	*	*	*	*	*	*		7 low
Rentsch CT et al [16]	*	*	*	*	**	*	*	*	9 high
Mehta N et al [17]	*	*	*	*	*	*	*	*	8 high
Mancia G et al [18]	*	*	*	*	**	*	*	*	9 high
de Lusignan S et al [19]	*	*	*	*	**	*	*	*	9 high
Huang Z et al [20]	*		*	*	*	*	*	*	7 low
Jung S-Y et al [21]	*	*	*	*	**	*	*	*	9 high
Li J et al [22]	*	*	*	*	**	*	*	*	9 high
Zhang P et al [23]	*	*	*	*	**	*	*	*	9 high
Richardson S et al [24]	*	*	*	*	**	*	*	*	9 high
Bean DM et al [25]	*	*	*	*	**	*	*	*	9 high
Tan N-D et al [26]	*	*	*	*	*	*	*	*	8 high
