# Supplementary material for: Does taking an angiotensin inhibitor increase the risk for COVID-19? – a systematic review and meta-analysis
Source: Aging (Albany NY). 2021 Apr 22;13(8):10853–65. doi: 10.18632/aging.202902 (PMC8109111; doi:10.18632/aging.202902)
Supplement: Supplementary Table 2 [file aging-13-202902-s003.docx]

Supplementary Table 2. Use of ACEIs or ARBs and odds of receiving a positive COVID-19 test result.

| Study | Period, population and country | Total number | Patients characteristics | Positive COVID-19 test | | Patients receiving ACEI/ARB | | | Patients not receiving ACEI/ARB | | |
| --- | --- | --- | --- | --- | --- | --- | --- | --- | --- | --- | --- |
|  |  |  | age, male | hypertension | Non-hypertension | positive COVID-19 test | Mortality in patients with positive COVID-19 | Severity cases in patients with positive COVID-19 | positive COVID-19 test | Mortality in patients with positive COVID-19 | Severity cases in patients with positive COVID-19 |
| Gnavi R et al [11] | 20/02/22-20/03/23;  First population(HY): 1896 hospital discharged(2015-2019), aged 40 years or older  Second population(CDD): drug presentation database in 2019, aged 40 years or older;  Italy | 2922 | HY: mean age 71.4 years with 78.4% male  CDD: mean age 74.5 years with 78.4% male | NA | NA | 308/1936 | NA | NA | 179/986 | NA | NA |
| de Abajo FJ et al [12] | 20/03/01-20/03/24;  Patients aged 18 years or older admitted to seven hospitals in Madrid, Spain | 12529 | 1139 cases: mean age 61.9 years with 61% male  11390 control: mean age 61.9 years with 61% male | 617/6261 | 522/6268 | 497/4319 | NA | NA | 642/8210 | NA | NA |
| Chodick G et al [13] | Maccabi Health Services database between Jan 1st,  2020 and date of first SARS-COV-2 test. | 14520 | 1317 positive SARS-COV-2: mean age 40.6 years with 58.9% male;  13203 negative SARS-COV-2: mean age 37.0 years with 46.1% | 185/1630 | 1132/12890 | 132/991 | NA | NA | 1185/13529 | NA | NA |
| Reynolds HR et al [14] | 20/03/01-20/04/15  Patients in Langone health system; United  States | 12594 | Mean age 49 years with 58.5% male | 2573/4357 | 3321/12890 | 1374/2319 | NA | NA | 4520/10275 | NA | NA |
| Yang G et al [15] |  | 2068 |  |  |  | 43/731 | 2/43 | 15/43 | 83/1337 | 19/208 | 51/208 |
| Rentsch CT et al [16] | 20/02/08-20/03/30;  Patients aged 54-75 years in the Veterans Health Administration;  United States | 3789 | Mean age 65.7 years with 90.2% male | 423/2463 | 162/1326 | 225/1532 | NA | NA | 330/2257 | NA | NA |
| Mehta N et al [17] | 20/03/08-20/04/12;  Patients within the Cleveland Clinic Health System in Ohio and Florida;  United States | 18472 | Mean age 49 years with 40%  male | 682/7312 | 1053/11160 | 214/2304 | 8/212 | 47/212 | 1521/16168 | 34/1527 | 762/1527 |
| Mancia G et al [18] | 20/02/21-20/03/11;  Residents 40 years or older in Lombardy, Italy | 37031 | Mean age 68 years with 63% male | 3632/18051 | 2640/18080 | 2896/15375 | NA | NA | 3376/21656 | NA | NA |
| de Lusignan S et al [19] | patients in the Oxford Royal College of General Practitioners Research and Surveillance Centre network;  United Kingdom | 3802 | Median age was 58.0 years for men and 51.5 years for women;  Male: 42.4% | 209/1094 | 378/2708 | NA | NA | NA | NA | NA | NA |
| Huang Z et al [20] | 20/02/07-20/03/03;  hospitalized hypertension patients;  Wuhan, China | 50 | RAS blockers group:  Mean age was 52.65,meal 50%  non-RAS blockers group: mean age was 76.77, male 56.7% | NA | NA | NA | 0/20 | 13/20 | NA | 3/30 | 24/30 |
| Jung S-Y et al [21] | As of 20/04/08;  Patients with positive COVID-19 test;  Republic of Korea | 5179 | Mean age was 44.6 years with 44% male | NA | NA | NA | 33/762 | NA | NA | 51/4417 | NA |
| Li J et al [22] | 20/01/15-20/03/15;  Patients hospitalized with  COVID-19;  Wuhan, China | 1178 | Median age was 55.5 years with 46.3% male | NA | NA | NA | 21/115 | 57/115 | NA | 109/1063 | 329/1063 |
| Zhang P et al [23] | 19/12/31-20/02/20;  Patients with hypertension and COVID-19 infection  admitted to nine hospitals;  Hubei, China | 1128 | ACEI/ARB group: median age 64 years with 53.2% male;  non-ACEI/ARB group: median age was 64 years with 53.5% male | NA | NA | NA | 7/188 | NA | NA | 22/940 | NA |
| Richardson S et al [24] | 20/03/01-20/04/04;  Hypertension patients with COVID-19 admitted to 12  Hospitals;  United States | 1366 | NA | NA | NA | NA | 130/413 | 87/413 | NA | 254/953 | 141/953 |
| Bean DM et al [25] | 20/03/01-20/04/13;  Acute inpatients  with COVID19 at two hospitals; London, United Kingdom | 1200 | Mean age was 68 years with 57% male | NA | NA | NA | 100/399 | 21/399 | NA | 182/801 | 106/801 |
| Tan N-D et al [26] | 20/01/28-20/04/08;  Hypertension patients with positive COVID-19;  Wuhan, China | 100 | ACEI/ARB group: mean age was 67 years with 54.8% male;  non-ACEI/ARB group: mean age 67.5 years with 52.1% male | NA | NA | NA | 0/31 | 27/31 | NA | 11/69 | 60/69 |

COVID-19, Corona Virus Disease 2019; ACEIs, Angiotensin-Converting Enzyme Inhibitors; ARBs, Angiotensin Receptor Blockers; NA, Not Available.
